# Occupational status and chronic respiratory diseases: a cross-sectional study based on the data of the Rafsanjan Cohort Study

**DOI:** 10.1186/s12890-024-02916-y

**Published:** 2024-03-23

**Authors:** Zahra Bagheri-Hosseinabadi, Ali Bahreyni, Hosein Basirat, Parvin Khalili, Alireza Vakilian, Fatemeh Amin

**Affiliations:** 1 Molecular Medicine Research Center, Research Institute of Basic Medical Sciences, Rafsanjan University of Medical Sciences, Rafsanjan, Iran; 2 Department of Clinical Biochemistry, School of Medicine, Rafsanjan University of Medical Sciences, Rafsanjan, Iran; 3 Medical student, Kerman University of medical Sciences, Kerman, Iran; 4 Student Research Committee, Rafsanjan University of Medical Sciences, Rafsanjan, Iran; 5 Social Determinants of Health Research Centre, Rafsanjan University of Medical Sciences, Rafsanjan, Iran; 6 Department of Epidemiology, School of Public Health, Rafsanjan University of Medical Sciences, Rafsanjan, Iran; 7 Non-Communicable Diseases Research Center, Rafsanjan University of Medical Sciences, Rafsanjan, Iran; 8 Neurology Department, School of Medicine, Rafsanjan University of Medical Sciences, Rafsanjan, Iran; 9 Physiology-Pharmacology Research Center, Research Institute of Basic Medical Sciences, Rafsanjan University of Medical Sciences, Rafsanjan, Iran; 10 Department of Physiology and Pharmacology, School of Medicine, Rafsanjan University of Medical Sciences, Rafsanjan, Iran

**Keywords:** Chronic respiratory diseases, Occupational status, Prospective epidemiological research studies in Iran (PERSIAN), Rafsanjan Cohort Study

## Abstract

**Background:**

The aim of the present study was to investigate the possible connection between occupational status and chronic respiratory diseases (CRDs) among the Iranian population.

**Methods:**

The present cross-sectional study was conducted on 9934 individuals aged 35–70 years enrolled in the Rafsanjan Cohort Study (RCS), a component of the Prospective Epidemiological Research Studies in Iran (PERSIAN). Detailed questionnaires were used to collect information on various factors, such as occupation, sociodemographic characteristics, medical history, anthropometric measurements, physical activity, cigarette and hookah smoking, opium use, and alcohol consumption. The association between occupational class and CRD was evaluated using logistic regression models for rare events.

**Results:**

In the present study, 4624 (46.55%) participants were male, and 5310 (53.45%) were female. The prevalence of CRD among all participants was 2.61%. Occupational activities were classified into two categories: In class I, the largest group was the homemaker and unemployment category (41.73%), followed by self-employment (34.39%), employment (13.03%), and retired individuals (10.84%). In class II, there were pistachio farmers (12.61%), copper miners (3.62%), and others in various occupations (83.76%). Subjects with CRD were significantly more likely to be homemakers, unemployed, elderly, female, less educated, and obese. There was no significant relationship between CRD and job type/occupational status after adjusting for some potential confounding variables.

**Conclusions:**

There was no significant relationship between CRD and job type/occupational status. However, longitudinal studies are needed to assess the impact of job type/occupational status on the risk of CRD.

## Introduction

Chronic respiratory diseases (CRDs) are a prevalent group of condition that primarily impact the lungs and airways. According to the World Health Organization (WHO), every year, 4 million people die from CRD, annually, with about 90% of these deaths occurring in low- and middle-income countries [1–3]. Chronic obstructive lung disease (COPD), occupational asthma, interstitial pulmonary disease, silicosis, asbestosis, inflammatory lung disease, and lung cancer are examples of respiratory diseases that significantly impact public health [4]. There are various risk factors that can contribute to the development of CRD, including allergens, tobacco smoking (including exposure to second-hand smoke), air pollution, occupational exposure, and frequent lower respiratory infections during childhood [3].

In developing and newly industrialized countries, industrial workers are often exposed to high levels of dust [5]. Exposure to hazardous airborne particles in the workplaces can lead to health risks. Air pollution can cause airway disorders, such as bronchial asthma and chronic obstructive pulmonary disease (COPD) [6, 7]. In fact, it is estimated that 15.6% of deaths related to COPD are caused by occupational exposure to airborne particulates [8]. Several studies have established a connection between occupational exposure to fumes, dust, and gases and the development of chronic bronchitis, and airflow obstruction. The underlying mechanism of the impact of occupational exposure on CRD is currently unknown. The challenge of establishing a clear relationship between occupational exposure and respiratory diseases is partly attributed to the difficultly of distinguishing the independent effects of smoking [9, 10]. However, some researchers have suggested that inhaled ultrafine particles interact with lung cells, such as epithelial cells and, alveolar macrophages, and lead to the production of reactive oxygen species. Reactive oxygen species (ROS) and calcium enter cells, stimulate the expression of proinflammatory genes (for example, cytokines) and cause inflammation [11–13].

In Iran, the significance of occupational health has been growing due to the hazards encountered by workers in diverse industries such as construction, mining, agriculture, manufacturing, and healthcare. Among these, farm workers are at a higher risk of being exposed to pesticides through inhalation and skin contact. They commonly exhibit a high prevalence of breathlessness, cough, phlegm, throat discomfort, chronic bronchitis, asthma, and allergic rhinitis [14, 15].

Therefore, identifying occupational respiratory diseases in the workplace and understanding the factors that affect them can be crucial for implementing effective strategies to enhance the health of workers and control the spread of these diseases, particularly among individuals exposed to infections or severe illnesses.

Accordingly, we decided to investigate the relationship between occupational status and CRD among a large population sample to mitigate the effects of pollutants, which are significant occupational hazards.

## Methods

### The study population

This cross-sectional investigation was conducted on participants enrolled in the Rafsanjan Cohort Study (RCS), a prospective epidemiological research project in Iran (PERSIAN) [16]. The RCS process was launched in August 2015 in Rafsanjan, southeast Iran. The study population included 9991 participants aged 35 to 70 years [16].  Among this population, individuals with completed medical history and occupation data were included in the present study. The study protocol was developed in accordance with the Persian cohort study and was approved by the Ethics Committee of Rafsanjan University of Medical Sciences.

### Definition and measurements

Participants were interviewed by trained interviewers using a standardized questionnaire that included inquiries about demographic information, socioeconomic status, occupational status, personal habits (such as smoking, hookah, opium, etc.), history of underlying diseases (such as diabetes, hypertension, kidney and liver disease, etc.), and animal contact. Additionally, anthropometric measurements were performed. The validity and reliability of these questionnaires have been confirmed in the Persian cohort study. Participants who reported being diagnosed with CRD, including chronic bronchitis, emphysema, asthma, tuberculosis, etc., by a physician were considered to have a positive history of this disease.

In terms of occupational status, individual who did not complete the questionnaire regarding their occupation or had uncertainty and/or multiple jobs were excluded from the study. Occupational status was classified according to the system of the Rafsanjan Cohort Profile and divided into two main categories of occupations (Classes I and II). Class I included the following subgroups: unemployed and homemaker, retired, self-employed, and employed. Class II, included pistachio farmers, copper miners, and others. Subjects in class II may overlap with those in class I, and in some cases, individuals had two jobs [17].

Education level was categorized into three groups: (1) no education or 1–5 years of education, (2) 6–12 years of education, and (3) more than 12 years of education. The personal habit data, including opium use and alcohol consumption, were expressed as “yes” (currently or formerly) and “no” (never), while cigarette smoking was expressed as “current”, “former” and “never” [16]. The prevalence of diabetes mellitus, cardiovascular diseases (CVD) and hypertension was assessed through a self-report questionnaire regarding medical history or medication. Body mass index (BMI) was calculated by dividing weight (kg) by height squared (m^2^) and was categorized as follows: BMI < 25, 25.0 ≤ BMI < 30, and BMI ≥ 30. The results were expressed as the mean ± standard deviation. The Wealth Score Index (WSI) was used to assess the socioeconomic status, and it was calculated through multiple correspondence analysis (MCA) of various economic and social variables. The intensity of physical activity was assessed using a 22-item questionnaire that measured 24-hour physical activity metabolic equivalent of task (MET).

### Statistical analyses

To describe the data, the mean and standard deviation for the quantitative variables were used, while frequency and percentage were used for categorical variables. Baseline characteristics of individuals were compared across the study groups (non-chronic respiratory disease, chronic respiratory disease) using chi-square (χ²) and t -tests for categorical and continuous variables, respectively. The association between occupational class and the odds of CRD was evaluated using logistic regression models for rare events (Stata command: firthlogit). Confounders were included in the models based on their assumed association with occupation class and CRD. Confounders such as age, sex, education, body mass index (BMI), WSI, physical activity, smoking, alcohol consumption, opium use, animal contact, history of diabetes, history of hypertension, and history of CVD were measured in separate models at the bivariate level. A *p*-value of ≤ 0.10 was considered for multivariate analysis. Basic sociodemographic specifications (occupation class, age, gender, and education) are included in adjusted model 1. Adjusted model 2 included all variables from adjusted model 1 as well as lifestyle confounding variables such as body mass index, physical activity, smoking, and history of CVD diseases. All analyses were performed using Stata V.12. All *p*- values are two-sided, and those less than 0.05, along with 95% confidence intervals, were considered statistically significant.

## Results

In this study, 9934 participants from the baseline phase of the Rafsanjan adult cohort study, who completed a medical questionnaire and provided job information, were included. From this population, 4624 (46.55%) were male and 5310 (53.45%) were female. The baseline characteristics of the subjects are presented in Table 1. The prevalence of CRD among all participants was 2.61%. individuals with CRD were significantly more likely to be homemakers, unemployed, elderly, female, less educated, and obese (BMI ≥ 30) (Table 1). Histories of CVD and former smoking were significantly higher among individuals with a history of CRD compared to those ease than among people without a history of CRD. There were no significant associations between a history of CRD and type of job, physical activity, WSI, use of opium, alcohol consumption, history of hypertension, diabetes, or animal contact (Table 1).

**Table 1:** Baseline characteristics of the Rafsanjan cohort participants according to CRD

Characteristics	Total (*n=*9934)	Chronic respiratory disease (*n*= 259)	No chronic respiratory disease (*n*=9675)	*P* Value
Job class 1.no. (%)				
Employment	1294(13.03)	25(9.65)	1269(13.12)	0.013
Homemaker and Unemployment	4145(41.73)	127(49.03)	4018(41.54)	
Retired	1077(10.84)	35(13.51)	1042(10.77)	
Self-employment	3416(34.39)	72(27.80)	3344(34.57)	
Job class 2.no. (%)				
Copper miner	361(3.63)	11(4.25)	350(3.62)	0.119
Pistachio farmer	1254(12.63)	22(8.49)	1232(12.74)	
Other	8317(83.74)	226(87.26)	8091(83.65)	
Age-year.no. (%)				
35-44	3697 (37.22)	72(27.80)	3625 (37.47)	0.002
45-54	3060 (30.81)	83(32.05)	2977(30.77)	
55-64	3176(31.97)	104(40.15)	3072(31.76)	
(Mean± SD)	49.94±9.56	51.76±9.56	49.89±9.56	0.002
Gender- no. (%)				
Male	4624 (46.55)	104(40.15)	4520 (46.72)	0.037
Female	5310 (53.45)	155(59.85)	51.55(53.28)	
Education-no. (%)				
<=5 years	3484 (35.09)	109(42.08)	3375 (34.90)	0.057
6/12 years	4820 (48.54)	112 (43.24)	4708 (48.69	
>=13 years	1625 (16.37)	38 (14.67)	1587 (16.41)	
(Mean± SD)	8.52±5.05	7.84±5.29	8.54±5.04	0.03
BMI- no. (%)				
<25	2868 (28.89)	60 (23.17)	2808 (29.04)	0.001
25-29.9	4070 (41.00)	95 (36.68)	3975 (41.11)	
≥30	2990 (30.12)	104 (40.15)	2886 (29.85)	
(Mean± SD)	27.82±4.92	28.58±5.22	27.80±4.91	0.01
Physical activity				
(Mean± SD)	38.79±6.32	38.12±6.60	38.81±6.31	0.082
WSI				
(Mean± SD)	-0.02±1	-0.047±1.13	-0.001±1	0.464
Use opium- no. (%)				
yes	2345 (23.65)	62 (24.03)	2283 (23.64)	0.885
no	7569 (76.35)	196 (75.97)	7373 (76.36)	
Alcohol consumption- no. (%)				
yes	993 (10.02)	19 (7.36)	974 (10.09)	0.151
no	8921 (89.98)	239 (92.64)	8682 (89.91)	
Cigarette smoking-no. (%)				
current	1679 (16.94)	24 (9.30)	1655 (17.14)	<0.001
never	7371 (74.35)	200 (77.52)	7171 (74.26)	
Former	864 (8.71)	34 (13.18)	830 (8.60)	
Hypertension- no. (%)				
yes	2235 (22.50)	68 (26.25)	2167 (22.40)	0.142
No	7699 (77.50)	191 (73.75)	7508 (77.60)	
CVD - no. (%)				
yes	1031(10.38)	45(17.37)	986(10.19)	<0.001
No	8903(89.62)	214(82.63)	8689(89.81)	
Diabetes mellitus- no. (%)				
yes	1933 (19.46)	58 (22.39)	1875 (19.38)	0.227
No	8001 (80.54)	201 (77.61)	7800 (80.62)	
Animal Contact no. (%)				
yes	8727 (87.96)	228 (88.03)	8499 (87.96)	0.974
No	1194 (12.04)	31 (11.97)	1163 (12.04)	

*Abbreviations*: *BMI *Body mass index, *WSI *Wealth score index, *CVD *Cardiovascular disease

Table 2 presents the association of occupational classes with CRD using the crude and two adjusted models. In the crude regression model, the odds of chronic lung disease were higher among the retired group (AOR: 1.70, 95% CI:1.01–2.84, *P*-value: 0.037) and the homemaker and unemployment group (AOR: 1.58, 95% CI:1.03–2.43, *P*-value: 0.045) compared to the employment group. However, this association was no longer significant after adjusting for confounding variables such as age, sex, years of education, smoking, physical activity, BMI, and CVD.

**Table 2:** Associations between occupational classes and chronic respiratory disease

	Crude model		Adjusted model 1		Adjusted model 2	
	AOR (95%Ci)^**a**^	***P*** -value	AOR(95%Ci)^**b**^	***P*** -value	AOR (95%Ci)^**c**^	***P*** -value
**Occupational classes I**						
Employment	1	-	1	-	1	-
Homemaker and Unemployment	1.58(1.03-2.43)	0.037	1.25(0.73-2.15)	0.411	1.23(0.71-2.11)	0.457
Retired	1.70(1.01-2.84)	0.045	1.35(0.78-2.33)	0.288	1.34(0.77-2.34)	0.295
Self-employment	1.08(0.68-1.70)	0.743	1.00(0.61-1.59)	0.963	1.02(0.63-1.66)	0.926
**Occupational classes II**						
Other	1	-	1	-	1	-
Farmer	0.65(0.42-1.01)	0.055	0.656(0.41-1.06)	0.086	0.67(0.41-1.08)	0.102
Miner	1.17(0.64-2.14)	0.605	1.26(0.67-2.37)	0.465	1.26(0.67-2.37)	0.479

^a^Baseline model is stratified by occupational classes

^b^Adjusted model 1 is adjusted for the confounding variables age (continuous variable), gender (male/female), and education (continuous variable)

^c^Adjusted model 2 has additional adjustment for the confounding variables smoking (current, former, never), physical activity (continuous variable), BMI (continuous variable) and CVD (yes/no)

In addition, in the crude model, the odds of CRD were lower among farmers (AOR: 0.65, 95% CI 0.42–1.01, *P*-value: 0.055) compared to the other job group. After adjusting for the variables (adjusted models 1 and 2), the results showed no significant association (Table 2).

## Discussion

The purpose of this study was to investigate the relationship between occupational status and CRDs in the population of Rafsanjan. The results indicated a significant relationship between CRDs and occupational status. The highest incidence of CRD was found among homemakers and unemployed individuals. However, no relationship was detected between CRD and job type/occupational status after adjustment for some potential confounding variables.

Indoor air pollution is responsible for numerous respiratory issues, as it contains various harmful substances that have been linked to an increased risk of respiratory complications [18]. In this study, the largest group in class II (41.73%) who may be exposed to indoor pollution were homemakers and unemployed individuals. Individuals with CRD were significantly more likely to be homemakers and unemployed, however, this association was no longer significant after adjusting for confounding variables. Golshan et al. demonstrated rural women doing indoor jobs in Iran are potential risk factors for development of chronic obstructive pulmonary disease [16]. There are various theories proposed to explain the connection between homemaking and respiratory diseases. These include indoor air pollution from sources like dust, cleaning products, and pets [18], as well as the potential impact of reduced physical activity and obesity [19].

Unemployed persons, in particular, are at a higher risk of respiratory diseases due to their low socio-economic status, marginalization in the labour market [20], mental health issue [21], living in areas with high environmental pollution, all of which can impact their respiratory health [22]. Yildiz et al. also reported that unemployed individuals have a higher prevalence of respiratory diseases than employed individuals [22].

Rafsanjan owns one of the largest pistachio orchards, covering with 80,000 hectares of agricultural land. In this study, 1254 pistachio farmers (12.63%) were found to be exposed to various occupational hazards, such as organic dust, inorganic dust, agrichemicals, allergens, toxic gases, and microorganisms. These exposures increase their susceptibility to respiratory problems [23]. Approximately 100,000 L of insecticides are used annually for pistachios cultivation in this city. There are four commonly used groups of insecticides: organophosphates (OP), carbamates (CBs), pyrethroids (PYs), and nicotinoids (N). All of these synthetic insecticides have the potential to cause significant health risks and various negative consequences [24, 25].

Previous studies have indicated that long-term workplace exposures could significantly increase the risk of CRD [26–28]. Lee et al. discovered that the total duration of pesticide spraying does not significantly affect the results of pulmonary function tests. However, a notable positive correlation was found between the use of mixed pesticides and an obstructive pattern, even after adjusting for confounding factors [29]. Melon et al. also reported that occupational exposure to vapors, dust, gases, and fumes is associated with a slight but increased risk of COPD [28]. Halvani et al. demonstrated a significant reduction in breathing capacity in the farmer group compared to non-farmers in Yazd, Iran [30]. Another study conducted in Iran showed a high prevalence of work-related respiratory symptoms in agricultural farmers, especially sheep breeders [31]. Conversely, workplace exposures in several studies showed low rates of reported respiratory symptoms. For instance, farmworkers in LatinX community in New York do not appear to exhibit any unusual respiratory symptoms or reduced breathing capacity [32]. Another study indicated that all farmworkers had FEV1/FVC ratios greater exceeding 70% of the predicted value, suggesting that their lung function was normal [33]. Consistent with these studies, there was no significant association between being a farmer and CRD in the present study. This may be because land-owning farmers hire workers to apply pesticides and other chemicals in agriculture, and they are not directly exposed to agriculture chemicals.

This city also boasts the second largest copper mine in the world. The presence of copper mines can lead to an increase in certain heavy metals, such as copper and arsenic, in the atmosphere. Inhaling these metals can have an impact on the respiratory system [34]. Additionally, inhaling other workplace pollutants, such as silica, coal, asbestos, organic dusts, mineral dusts, oxides of nitrogen, sulfur dioxide, and other gas and fume contaminants, has been linked to with disabilities and work-related respiratory diseases [35]. Although copper (Cu) is recognized as an antioxidant cofactor in the body, a study has indicated that elevated serum Cu levels in copper mining workers are significantly negatively correlated with lung function [36]. Gholami et al. also reported that exposure to dust in four industries in Iran has a detrimental role in respiratory function [37]. However, the present study, found no relationship between being a copper miner and CRD. The discrepancy in the results may be due to the fact that the individuals who took part in the study as copper miners were involved in administrative, laboratory, transportation, and other areas with lower exposure to heavy pollutants compared to mine workers.

Smoking is recognized as the primary risk factor for respiratory disease, accounting for over 75% of cases. Previous studies have shown that 80 to 90% of patients with COPD have a history of smoking [38]. The findings of the current study, consistent with prior research, demonstrate a significant association between smoking and respiratory disease. This suggests that smoking can be a primary factor in chronic respiratory diseases, even in the absence of occupational exposures. Based on our results, 23.81% of RCS participants used opium at least once a week for 6 months [17]. We argue that drug abuse is probably an important risk factor in the present study, which naturally increases the chance of respiratory diseases, along with higher consumption of cigarettes and alcohol by affected individual.

In this study, we observed that the prevalence of CVD was significantly higher among people with a history of CRD compared to those among people without a history of CRD. Additionally, another study revealed that people with chronic obstructive pulmonary disease often experience cardiovascular diseases, leading to an increased risk of hospitalization, longer stays, and higher mortality rates related to all causes and cardiovascular diseases [39].

The study had notable strengths, including a large sample size, population-based research, and careful consideration of several confounding variables. On the other hand, the cross-sectional design of the study limited the ability to establish causal relationships, which will be investigated in the prospective follow-up phase of the study. Another limitation of this study was the lack of available information regarding the previous job history of the cohort, particularly for elderly and unemployed individuals. Additionally, we were unable to assess the extent of exposure to occupational contaminants or the specific occupational pollutants to which they were exposed. Furthermore, individuals in certain professions may not have been initially exposed to pollutants as we assumed. For instance, a farmer may not have used pesticides on their crops or may have assigned such tasks to laborers, while a miner may have worked in a managerial role rather than being directly involved in the mining process. These factors could have influenced on our findings and should be considered in future research.

This study provides valuable insights for policymakers and regulatory authorities, suggesting the potential implementation of public education programs by relevant experts to reduce exposure to potential respiratory hazards for farmers, miners, and homemakers in their workplace. Furthermore, policy guidelines should consider previous exposure to occupational pollutants and lifestyle factors. It is essential to inform results of this study to the decision-makers for planning to reduce the incidence and mortality of CRD. In addition, future research should explore additional potential risk factors for CRD beyond job type and occupational status, such as genetic factors, etc.

## Conclusion

There was no association between CRD and job type/occupational status after adjusting for some potential confounding variables. However, longitudinal studies are needed to assess the effect of job type/occupational status on the risk of CRD. The novelty of this study lies in its unique focus on the specific occupational and environmental factors present in Rafsanjan, particularly the impact of agricultural toxins and copper mine pollution on respiratory health. This study represents the first large-scale investigation into the relationship between occupational status and chronic respiratory disease within this specific context. The findings provide valuable insights into the potential effects of these occupational and environmental factors on respiratory health, paving the way for future research and follow-up studies to confirm and build upon these initial results. This study’s contribution is particularly significant in shedding light on the intersection of occupational status, environmental exposure, and respiratory health within the context of Rafsanjan, offering valuable knowledge for both researchers and public health practitioners.

## Data Availability

The data is not publicly accessible, but it can be obtained from the corresponding author upon a reasonable request.
